# Differences in plasma levels of long chain and very long chain ceramides between African Americans and whites: An observational study

**DOI:** 10.1371/journal.pone.0216213

**Published:** 2019-05-08

**Authors:** Joy N. Jones Buie, Samar M. Hammad, Paul J. Nietert, Gayenell Magwood, Robert J. Adams, Leonardo Bonilha, Catrina Sims-Robinson

**Affiliations:** 1 WISSDOM Center, Medical University of South Carolina, Charleston, SC, United States of America; 2 Department of Neurology, Medical University of South Carolina, Charleston, SC, United States of America; 3 Department of Regenerative Medicine and Cell Biology, Medical University of South Carolina, Charleston, SC, United States of America; 4 Department of Public Health Sciences, Medical University of South Carolina, Charleston, SC, United States of America; 5 College of Nursing, Medical University of South Carolina, Charleston, SC, United States of America; University of Southern Queensland, AUSTRALIA

## Abstract

**Background:**

Population-wide reductions in cardiovascular disease (CVD) have not been equally shared in the African American community due to a higher burden of CVD risk factors such as metabolic disorders and obesity. Differential concentrations of sphingolipids such as ceramide, sphingosine, and sphingosine 1-phosphate (S1P) has been associated with the development of CVD, metabolic disorders (MetD), and obesity. Whether African Americans have disparate expression levels of sphingolipids that explain higher burdens of CVD remains unknown.

**Methods:**

A cross sectional analysis of plasma concentrations of ceramides, sphingosine, and S1P were measured from 8 whites and 7 African Americans without metabolic disorders and 7 whites and 8 African Americans with metabolic disorders using high performance liquid chromatography/tandem mass spectrometry methodology (HPLC/MS-MS). Subjects were stratified by both race and metabolic status. Subjects with one or more of the following physician confirmed diagnosis: diabetes, hypertension, hypercholesterolemia, or dyslipidemia were classified as having metabolic disease (MetD). Data was analyzed using a Two-Way ANOVA and Tukey’s post hoc test.

**Results:**

Total ceramide levels were increased in African Americans compared to African Americans with MetD. Ceramide C16 levels were higher in whites with MetD compared to African Americans with MetD (p<0.05). Ceramide C20 levels were higher in whites with MetD compared to whites. Ceramide C20 levels were higher in African Americans compared to African Americans with MetD. Furthermore, whites with MetD had higher levels of C20 compared to African Americans with MetD (p<0.0001). Ceramide C24:0 and C24:1 in African Americans was higher compared to African Americans with MetD (p<0.05). The plasma concentration of Sph-1P ceramide was higher in African Americans vs whites (p = 0.01). Lastly, ceramide C20 negatively correlated with hemoglobin A1c (HbA1c) levels in our study cohort.

**Conclusions:**

Plasma ceramide concentration patterns are distinct in African Americans with MetD. Further research with larger samples sizes are needed to confirm these findings and to understand whether racial disparities in sphingolipid concentrations have potential therapeutic implications for CVD-related health outcomes.

## Introduction

African Americans have not equally benefited from population-wide cardiovascular disease (CVD) prevention programs and are still twice as likely to die from heart disease compared to non-Hispanic whites [1–3]. Significant disparities in age of onset and the prevalence of hypertension, diabetes, and obesity underscore a higher burden of CVD and coronary heart disease (CHD) in African Americans [4–7]. Despite the increased burden of atherosclerotic CVD and coronary heart disease (CHD) in African Americans, reports on the prevalence of dyslipidemia in African Americans are conflicting with African Americans <45 years old having lower levels of low-density lipoprotein (LDL) cholesterol compared to non-Hispanic whites [2, 5, 8].

Ceramides are present in all cell types and lipoproteins including LDL [9]. During dyslipidemia and metabolic dysfunction, such is common with continuous consumption of the Southern style diet [10], ceramides are synthesized *de novo* and accumulate in tissues not suitable for lipid storage [11]. Unlike cholesterol which is mainly inert, ceramides have biological activity and its accumulation in plasma has been implicated in atherosclerotic plaque formation, endothelial dysfunction, hypertension, and insulin resistance that play a role in the development of CVD [12–17]. Recently, Meeusen et al. showed that plasma ceramide concentration scores serve as independent predictors of cardiovascular events in patients with normal LDL-C of <100mg/dL and in patients without coronary artery disease [18]. Moreover, ceramides serve as predictors of cardiovascular death in patients with stable coronary artery disease and acute coronary syndromes independent of LDL [19]. Thus, ceramide may serve as a better predictor of CVD risk in African Americans without dyslipidemia. However, studies examining distinct ceramide plasma concentrations and the predictive value of ceramides in CVD risk in African Americans are missing.

Ceramide-mediated insulin resistance is an important pathophysiological mechanism leading to poor CVD outcomes. Several studies have demonstrated the biological underpinnings of disrupted ceramide metabolism in the development of tissue specific insulin resistance [9, 20]. Data from the FINRISK study demonstrated that higher ceramide C18:0/C16:0 ratio was predictive of incident diabetes even after adjusting for BMI, fasting glucose, and hemoglobin A_1c_ [21]. Moreover, others have shown that C24:0/C16:0 and C22:0/C16:0 ceramide ratios are associated with all-cause mortality in a cohort of patients at risk for CVD [22]. However, these studies did not examine racial differences in ceramide levels to determine whether or not the predictive value of these ceramide biomarkers was applicable across different racial groups.

Despite efforts to eliminate racial disparities in cardiovascular health, little has changed over the past two decades [23]. While patient, provider, and system-related factors contribute to this disparity, factors such as socioeconomic risk factors do not fully explain the disparity in CVD and stroke [23, 24]. Given that African Americans are at increased risk for the development of diabetes and CVD, it is essential to understand ceramide biosynthesis and metabolism in this population compared to whites. To our knowledge, only one study has examined racial differences in ceramide levels [25] and has shown that African Americans have reduced levels of very long chain ceramides after adjusting for age and sex [25]. Further understanding racial variability in ceramides will aid in the development of prediction models to reduce racial health disparities in CVD. In the current study, we examined how ceramides differ in African Americans and whites with and without the presence of metabolic diseases.

## Materials and methods

### Ethics statement

The study was approved by the ethics committee of the Medical University of South Carolina and was performed according to the protocols approved by our institutions’ institutional review board (IRB). Prior to enrollment in the study, written informed consent was obtained from each subject.

### Subjects

We recruited 60 community dwelling subjects, who were between the ages of 30–75 years of age, without a history of stroke, as part of the American Heart Association/American Stroke Association Strategically Funded Research Network funded Wide Spectrum Investigation of Stroke Outcome Disparities on Multiple Levels (WISSDOM) study [26]. All subjects self-reported that they were not currently pregnant (if female) and did not have a history of dementia, a diagnosis of substance abuse, or the presence of a terminal illness with life expectancy of ≤ 1 year. This self-reported data was confirmed via medical chart review. Subjects with one or more of the following physician confirmed diagnosis: diabetes, hypertension, hypercholesterolemia, or dyslipidemia were classified as having metabolic disease (MetD). A third party stratified subjects based on race and metabolic status to ensure the experimenter remained blinded to the groups. Plasma samples were obtained from 59 of the 60 subjects resulting in 18 whites and 15 African Americans without MetD (healthy) and 14 whites and 12 African Americans with MetD (unhealthy). Plasma samples contaminated with blood due to cell lysis were excluded from this study. This pilot study was focused on a subset of subjects randomly selected within each of the stratified groups, which included 25 women (mean age 54.5±7.5) and 6 men (mean age 58.6±9.6) for the examination of plasma lipid levels. Hence, this study consisted of 4 groups: 8 whites and 7 African Americans without MetD and 7 whites and 8 African Americans with MetD. Demographic characteristics including age, gender, smoking status, body mass index (BMI), blood pressure, and the use of medications for hypertension and hypercholesterolemia for the subjects in this pilot study are included in Table 1.

**Table 1:** Demographics of subjects.

	White	African Americans	*P-Value*	White-MetD	African Americans -MetD	*P-value*
Variable						
**Age, mean (SD)**	55.8 (9.5)	48.4 (5.6)^a^	*ns*	56 (4.5)	61.9 (5.7)^a^	*ns*
**Female, n (%)**	6 (75%)	7 (100%)	*ns*	4 (57%)	7 (87.5%)	*ns*
**Ever Smoker (%)**	4 (50%)	0 (0%)	*p<0*.*05*	5 (71%)	2 (25%)	*ns*
**Body Mass Index (SD)**	27.7 (5.0)	33.3 (7)	*ns*	29 (5.0)	32.3 (5.8)	*ns*
**Hypertension, n (%)**	0 (0%)	0 (0%)^b^	*ns*	3 (43%)	7 (88%)^b^	*p<0*.*001*
**SBP, mean mmHg (SD)**	126.3 (12.7)	125.9 (16.6)	*ns*	121.3 (18)	135.6 (25.1)	*ns*
**DBP, mean mmHg (SD)**	75.9(5.1)	69.57 (7.1)	*ns*	74.6 (3.7)	73.9 (13.8)	*ns*
**Type 2 Diabetes, n (%)**			*ns*			*p<0*.*01*
*Diabetes*	0 (0%)	0 (0%)		0 (%)	3 (37.5%)	
*Diabetes with organ damage*	0 (0%)	0(0%)		1 (14.5%)	2 (25%)	
**Hypercholesterolemia, n (%)**	0 (0%)^c^	0 (0%)^d^	*ns*	6 (85.7%)^c^	4 (50%)^d^	*ns*
**Hypertensive Medication Use, n (%)**	0 (0%)^e^	0 (0%)^f^	*ns*	3 (43%)^e^	7 (88%)^f^	*ns*
**Cholesterol Lowering Medication Use, n (%)**	0 (0%)	0 (0%)	*ns*	5 (71%)	5 (56%)	*ns*
**Education**			*ns*			*ns*
*GED*, *High School Diploma or less*	1 (12%)	3 (42.9%)		2 (28.6%)	4 (50%)	
*At least some college*	7 (88%)	4 (57.1%)		5 (71.4%)	4 (50%)	
**Employment Status, n (%)**			*ns*			*ns*
*Employed*	5 (62.5%)	5 (71.4%)		5 (71.5%)	3 (37.5%)	
*Not Employed*	3 (37.5%)	2 (28.6%)		2 (28.5%)	5 (62.5%)	
**Income, n (%)**			*0*.*003*			*ns*
*Less than $50*,*000*	2 (25%)	7 (100%)		4 (57.1%)	7 (88%)	
*$50*,*000 or more*	6 (75%)	0 (0%)		3 (42.8%)	1 (12%)	
**Marital Status, n (%)**			*p<0*.*001*			*ns*
*Unmarried*	1(12%)	7 (100%)		3 (42.9%)	7 (88%)	
*Married*	7 (88%)	0 (0%)		4 (57.1%)	1(12%)	

The p-values are for comparisons between whites and African Americans with or without MetD. Significant differences within the same racial group are denoted by a letter. DBP, diastolic blood pressure; SBP, systolic blood pressure; NS, not significant; GED; Graduate Education Development certificate.

a; p = 0.001 for differences in age between African Americans groups

b; p<0.001 for differences in hypertension between African Americans groups

c; p<0.001 for differences in hypercholesterolemia/hyperlipidemia between white groups

d; p<0.001 for differences in hypercholesterolemia/hyperlipidemia between white groups

e; p<0.05 for differences in hypertensive medication use between white groups

f; p<0.01 for differences in hypertensive medication use between African Americans groups

### Blood sample collection

Blood was collected in BD Vacutainer tubes containing ethylenediaminetetraacetic acid (EDTA; #367842, lavender closure, Becton Dickinson, Franklin Lakes, NJ). Immediately following collection blood tubes were centrifuged at 4°C at 1000 RPM for 15 minutes. The plasma was stored at -80°C in 500-μl aliquots until lipid extraction and lipidomics analysis.

### Lipid extraction

Plasma sphingolipids were extracted and levels of sphingoid bases and their phosphates and ceramide species were measured at the Lipodomics Shared Resource Core Facility at the Medical University of South Carolina as previously described [27]. Plasma samples from each subject were thawed at 4°C for 2h and 100μl plasma sample was extracted and analyzed. Internal standards, supplied either by the Lipidomics Core or purchased commercially (Avanti Polar Lipids, Alabaster, AL; and Matreya LLC, Pleasant Gap, PA) with a purity ≥ 98%, were added to each sample for fortification (1,000pmol/ml each in methanol, 5,000 p/mol for SM; 50μl). Lipid samples were extracted with 2 ml of isopropanol and ethyl acetate (15:85 v/v) extraction solution, vortexed, and centrifuged for 5 min at 3,000 rpm on a Beckman Allegra 6R Centrifuge (Beckman Coulter, Brea, CA). The upper organic phase was transferred to a separate 8 ml glass tube. The remaining diluted plasma was acidified with 100 ml formic acid (98%) and an additional 2 ml of extraction solution was added to further facilitate completion of extraction. The samples were vortexed and centrifuged for 5 min at 3,000 rpm (2,500g). The upper organic phase was transferred and added to the glass tube containing the initial extract (total 4 ml extract) and vortexed. Extract was evaporated to dryness with an N-Evap 112 Nitrogen Evaporator (Organomation Associates, Berlin, MA). The dried residues were reconstituted in 150 μL HPLC mobile phase B (see composition of mobile phases below). The reconstituted samples were temporarily placed at 4°C in high-performance liquid chromatography (HPLC) auto-sampler screw-cap vials until ready for injection on the HPLC/MS/MS system.

### Sphingolipid analysis by high performance liquid chromatography mass spectrometry (HPLC-MS/MS)

The Lipidomics Core performed Quantitative sphingolipid analysis using HPLC-MS/MS as previously described [27]. Briefly, chromatographic separations were obtained under a gradient elution as previously described [28] using a mobile phase with ammonium formate formic acid in water and methanol on an Ascentis C8 HPLC column (Sigma-Aldrich, St. Louis, MO). Sphingolipids analyzed included Cn- of ceramide and sphingoid bases (C18:1, C18:0), sphingosine (Sph) and dihydrosphingosine (dhSph), and their phosphate derivatives sphingosine 1-phosphate (Sph-1P) and dihydrosphingosine 1-phosphate (dhSph-1P), as well as dihydro-C16 (dhC16) ceramide. Quantitative analysis were based on eight-point calibration curves generated for each available target analyte. The synthetic standards and a set of internal standards were spiked into an artificial matrix and subjected to an identical extraction procedure as the biological samples. These extracted standards were analyzed by the HPLC/MS/MS system operating in positive MRM mode employing a gradient elution. Peaks for the target analytes and internal standards were recorded and processed using the instrument’s software system. Plotting the analyte/internal standard peak area ratios against analyte concentrations generates the analyte specific calibration curves. Any sphingolipid for which no standards were available were quantitated using the calibration curve of its closest counterpart. The data was normalized to the volume of sample used. The unadjusted total plasma ceramide mean concentrations were calculated by adding all of the measured ceramides together for each individual plasma sample.

### Statistical analysis

The demographic and clinical characteristics were analyzed using SPSS (IBM, Cary, NC). All other statistical analysis was performed using GraphPad Prism 8.0 (San Diego, CA). Racial differences and metabolic disorder differences were examined using Fisher’s exact test for categorical variables and 2-sample t-test for continuous variables. Significant differences in ceramide concentrations among all four groups (whites, African Americans, whites MetD, and African Americans MetD were evaluated using a Two-Way ANOVA followed by a Tukey’s post-hoc analysis. We stratified groups based on race and metabolic disorder status due to the literature indicating differences in ceramide concentrations in relation to cardiovascular disease risk. If all groups passed the D’Agostino & Pearson normality test, then correlations were performed using the Pearson’s correlation (r) coefficients; however, if some of the groups failed the normality test then the nonparametric Spearman correlation (ρ) were used. Due to the small sample sizes, we were unable to adjust for potential confounders but did compare demographic data to examine differences in income, education level, marriage status, and employment status (Table 1). Data are expressed as mean ± standard error of the mean (SEM). All analyses significance was determined using an alpha-level of 0.05.

## Results

### Demographic and clinical characteristics of subjects

The concentrations of various sphingolipid species were measured in plasma samples collected between 2015 and 2016 from 30 subjects enrolled in the WISSDOM study. Characteristics of African Americans and white study subjects with and without metabolic disorders are summarized in Table 1. The present analysis included 24 women (mean age 54.9±7.4) and 6 men (mean age 58.6 ±9.6). Participant characteristics by race and metabolic disorder status are outlined in Table 1. There were 8 whites and 7 African Americans without MetD and 7 whites and 8 African Americans with MetD. Compared to whites with MetD, the African Americans MetD was older (mean: 61.9 vs 56, *p<0*.*001*). Accordingly, a higher proportion of the African Americans MetD group had a diagnosis of hypertension (88% vs 43%, p<0.001) and diabetes with or without organ damage (62.5% vs 14.5%, p<0.01) while whites were more likely to have a diagnosis of hypercholesterolemia (86% vs 50%) but this difference was not significant. Subjects with MetD were more likely to take anti-hypertensive medications compared to subjects without MetD (0% vs 71% and 0% vs 56%; *p<*0.01 and *p<*0.01 for white and African Americans, respectively). Whites without MetD were more likely to have smoked compared to African Americans (50% vs. 0%, *p<*0.05). Overall, whites had higher incomes than African Americans (75% ≥$50,000 vs 0% ≥$50,000, *p = 0*.*003*) the difference in income between whites and blacks with MetD was not significant. Whites without MetD were also more likely to be married compared to African Americans without MetD (88% vs 0%, *p<0*.*001*. There were no significant differences in education or employment between any of the groups.

### Modifications in circulating ceramide concentrations based on race and metabolic disorder status

The unadjusted mean concentrations of plasma sphingolipids measured in plasma samples ascertained from white and African American study subjects with and without MetD are summarized in Table 2. Total plasma ceramide levels were significantly lower in study participants who were African Americans with MetD compared to concentrations in African Americans without MetD (615.8± 92.8 *pmol*/ml vs. 441.6± 125.8 *pmol*/ml; *p =* 0.0233) and whites with MetD (609.6 ± 117 *pmol*/ml vs 441.6±125.8 *pmol/*ml; *p =* 0.041). Of the long chain ceramides, C16-ceramide was significantly elevated in whites with MetD compared to African Americans with MetD (85.4 ± 29.7 *pmol*/ml vs. 64.6 ± 33.6 *pmol*/ml, *p* = 0.0313). However, there were no significant differences in ceramide C14-, C18-, and C18:1-ceramide amongst the four study groups. Amongst very long chain ceramides, we observed that ceramide C20-ceramide concentrations were higher in whites with MetD compared to whites without MetD (123.1± 64.2 *pmol*/ml vs. 81.0 ± 21.9 *pmol*/ml, *p =* 0.0002). Notably, African Americans with MetD had significantly lower plasma concentrations of C20-ceramide in comparison to African Americans without MetD (91.1 ± 32.6 *pmol*/ml vs. 54.8 ± 28.1 *pmol*/ml, p = 0.0003) and whites with MetD (123.1± 64.2 vs. 54.8 ± 28.1 *pmol*/ml, *p<*0.0001). Plasma levels of C24:0 and C24:1-ceramide were lower in African Americans with MetD compared to African Americans without MetD (70.0 ± 20.2 *pmol*/ml vs 103.0 ± 26.2 *pmol*/ml, *p =* 0.0026) and (114.8 ± 30.6 *pmol*/ml vs 155.5± 30.5 *pmol*/ml, *p =* 0.0026), respectively. There were no significant differences in the concentration levels of other very long chain ceramides.

**Table 2:** Group differences in plasma sphingolipids stratified by MetD status and race.

Plasma Lipid	White (n = 8) mean (range)	African Americans (n = 8) mean (range)	White MetD (n = 7) mean (range)	African Americans MetD (n = 8) mean (range)
**Ceramide**				
**C14:0**	1.2 (1–1.5)	1.2 (0.7,1.6)	1.4 (0.7–1.8)	0.9 (0.5–1.8)
**C16:0**	76.4 (51.7, 97.8)	76.5 (62.5, 94.2)	85.4 (39.3, 129.3)	64.6 (30.1, 118,3)^b^
**C18:0**	32.26 (22, 61.5)	42.3 (27.7, 60.4)	37.2 (21.2, 57.2)	32.18 (17.2, 52.2)
**C18:1**	11.58 (6.5, 20,2)	20.1 (8.1, 37)	15.2 (2.4, 27.6)	13.3 (5.5, 20.1)
**C20:0**	81.0 (42.3, 114.4)	91.1 (52.5, 138)	123.1 (51.7, 239.6)^a^	54.8 (28.2, 115.3)^a^^b^^c^
**C20:1**	17.1 (12.1, 29.7)	20.0 (11.1, 28.6)	22.3 (9.4, 44.5)	16.8 (11.7, 30.1)
**C20:4**	0.4 (0, 0.6)	0.5 (0.1, 0.9)	0.4 (0, 1.7)	0.5 (0.1, 1.4)
**C22:0**	62.0 (47.3, 87.3)	72.1 (50.3, 90.7)	71.3 (47.6, 98.9)	52.4 (38.7, 79.1)
**C22:1**	18.91 (12.7, 27.4)	23.9 (17.9, 29.3)	23.0 (13.6, 33.9)	15.9 (9.7, 27.8)
**C24:0**	92 (53.8, 144.2)	103 (48.1, 130.8)	92.17 (69.1, 103)	70 (46.1, 102.6)^c^
**C24:1**	138.4 (81.6, 220.1)	155.5 (120, 211.8)	131.4 (101.7, 145.2)	114.8 (71.2, 150)^c^
**C26:0**	5.1 (2, 10.3)	4.8 (3.1, 7.4)	4 (2.1, 5.7)	2.9 (1.8, 4.7)
**C26:1**	3.3 (1.6, 5.9)	4.8 (2.6, 11.9)	2.7 (1.3, 3.6)	2.4 (1.4, 3.7)
**dHC16**	8.2 (1.7, 29.5)	13.4 (2.8, 22.7)	9.3 (2.1, 14.6)	4.6 (1.9, 13.4)
**Total Ceramides**	539.7(396.2, 749.4)	615.8 (524.9, 774.7)	609.6 (426, 744.4)	441.6 (338.3, 640.4)^b^^c^
**Sphingosine**				
**Sph**	3.8 (2.3, 6.6)	4.2 (3.2, 5.7)	3.6 (2.1, 5.6)	4 (2.6, 6.2)
**Sph-1P**	58.4 (20.6, 96.2)	88.9 (40.5, 132.8)^a^	65 (27.6, 101)	65.5 (39.8, 94.2)
**dh-Sph**	3.1 (1.2, 6.7)	2.3 (1.5, 4.2)	2.5 (1.2, 4.0)	3.3 (1.7, 10)
**dhSph-1P**	9.4 (1.9, 22.6)	17.7 (3.9, 33)	11.4 (2.2, 21.4)	10.5 (3.6, 15.1)

^a^ p<0.05 vs whites

^b^ p<0.05 vs whites with MetD

^c^p<0.05 vs African Americans, MetD; Metabolic Disorder

### Differential expression of sphingosine bases and their phosphates

Ceramides have several derivatives including sphingosine. We analyzed changes in sphingosine and sphingosine derivatives associated with metabolic diseases and insulin resistance in our study cohort. Sphingosine-1-phosphate (Sph-1P) was significantly higher in African Americans compared to whites and African Americans without MetD, (88.7 ± 29.53 vs. 58.4 ± 26.9, *p* = 0.0114 and 88.7 ± 29.53 vs. 65.5 ± 18.4), respectively. There were no differences in sphingosine or other sphingosine derivatives.

### Ceramide species are associated with a parameter of insulin resistance

**Fig 1:**
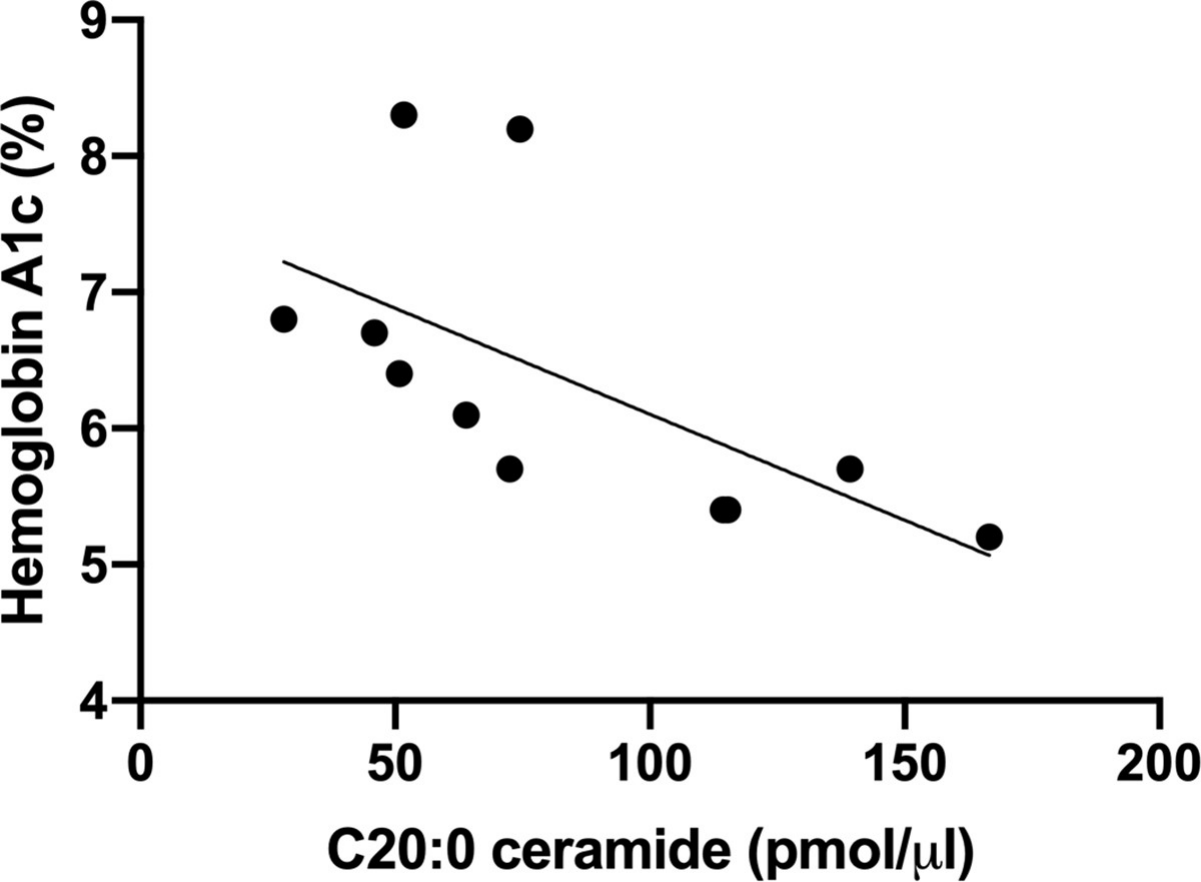
Differences in plasma levels of ceramide species among the groups. Correlation of C20:0 ceramide and hemoglobin A1c (%) from all groups.

It is well established that fluctuations in ceramide concentrations coincide with other parameters of metabolic dysfunction. To investigate the association of plasma ceramides and sphingosine with different parameters of metabolic dysfunction, we examined the correlation between the levels of ceramide or sphingosine/ sphingosine derivative molecular species and hemoglobin A1c, BMI, and creatinine. The plasma concentration of C20 ceramide correlated with hemoglobin A1c values (r = -0.6325; *p* = 0.0273; Fig 1). All other ceramides did not coincide with hemoglobin A1c levels. Subsequently, we examined the correlation between C20 and other plasma ceramides and found that C20 correlated with C22 and C22:1. Notably, we did not identify any ceramide correlates with BMI or serum creatinine concentrations.

## Discussion

Information is limited regarding racial differences in plasma sphingolipid concentrations. In the present study, we examined the race and MetD status dependent trajectories of plasma ceramides in subjects age 35–75. We observed higher sphingosine-1-phosphate concentrations in African Americans versus whites without MetD. The results showed that in African Americans with MetD, total ceramide plasma concentrations were significantly lower compared to African Americans and whites with MetD and concentrations of ceramide C:20 were significantly lower compared to all other groups. The results further revealed that two ceramide species- C24:0 and C24:1 were lower in African Americans with MetD vs African American controls. In comparison with whites with MetD, ceramide C16:0 levels were significantly lower in African Americans with MetD. The findings suggest that the race/MetD interaction may influence ceramide concentrations in the plasma. The results also point to the possibility that unique factors associated with the social construct of race might impact circulating ceramide levels. Collectively, these data suggest that factors associated with race may serve as important parameters when identifying biomarkers and therapeutic targets for the prevention of cardiovascular disease, metabolic disease and insulin resistance. These data, however, must be interpreted with caution as we were unable to adjust for potential confounders such as hypertension, diabetes, income, marital and smoking status due to small sample sizes.

Several cross-sectional studies have reported the association between metabolic disorders, plasma ceramide or dihydroceramide species [25]. Typically, higher ceramide concentrations are indicative of metabolic abnormalities including insulin resistance and obesity and tend to correlate with the severity of the disease [17]. In the present study, we found that African Americans with MetD had higher rates of diabetes and hypertension compared to whites with MetD. However, total plasma ceramide concentrations were lower and C16:0, commonly associated with insulin resistance, was decreased amongst African Americans with Met D as well. The reason for this paradox in plasma ceramide concentrations is unknown. In a large study examining plasma ceramides in 867 whites and 125 African Americans, most plasma ceramides were reduced in African Americans compared to whites [25]. Moreover, the authors showed that African Americans had less age-related increases in ceramides after controlling for potentially confounding variables including education, BMI and statin use. Notably, we ascertained the impact of race on plasma ceramide concentrations in subjects with and without metabolic disorders that have been previously shown to affect ceramides such as diabetes [29], hypercholesterolemia [3], and hypertension [15]. One possible explanation for this is the finding that sphingomyelin concentrations, are significantly higher in African Americans compared to whites [30]. Sphingomyelin plasma concentrations positively correlate with cholesterol levels [30] and sphingomyelin hydrolysis gives rise to ceramide and phosphocholine [31]. Sphingomyelinase activity is modulated by angiotensin II and recent studies suggest that losartan, an angiontensin receptor blocker, prevents sphingomyelinase activity in isolated carotid arteries [14]. The majority of African Americans with MetD within our study cohort were on anti-hypertensive medications which might provide a plausible explanation for lower total ceramide concentrations compared to African Americans. However, we did not adjust for hypertensive medication use due to the small sample size nor did we ascertain sphingomyelin concentrations in this study.

Although the data presented herein are exploratory and associative in nature, there are several novel findings that provide additional opportunities of exploration regarding the role of ceramides in African Americans with MetD that contradict the current ‘dogma’ of ceramide biology and theory. First, overexpression of CerS6 is known to enhance long-chain C16:0 ceramide generation and promote the development of type 2 diabetes and insulin resistance in mice and humans [32]. C16:0-ceramide impairs insulin signaling by antagonizing insulin-receptor mediated phosphorylation of PI3K/Akt leading to reductions in glucose metabolism. Therefore, reducing CerS6 could inhibit obesity induced CerS6 overexpression, leading to improved insulin sensitivity and reductions in metabolic disorders. Whether African Americans have altered CerS6 leading to subsequent reductions in C16:0-ceramide is unknown. Alternatively, overexpression of CerS2 and subsequent increases in very long-chain sphingolipid species have been shown to improve hepatocyte insulin sensitivity through insulin-induced phosphorylation of the insulin receptor (Tyr1150/1151) and Ser473 phosphorylation of Akt [33]. While CerS2 knockout animals fed a high fat diet, similar to the western diet, display reductions in the C24 series, upregulation of C16:0-ceramide and subsequent impaired glucose tolerance accompanied by high insulin concentration and loss of insulin sensitivity [34]. Very-long chain unsaturated ceramide species have also been associated with reduced-body fat and negatively correlate with visceral and liver fat in in human studies [32, 35]. Thus, reduced levels of very long chain ceramides could reflect unhealthy levels of adiposity/metabolic phenotype and could be contributing to poorer outcomes in African American patients with MetD by augmenting the insulin signaling pathway.

Whether African Americans have CerS2 polymorphisms that lead to impaired very-long chain ceramide synthesis remains to be studied. Higher total ceramide levels have been linked to insulin resistance, diabetes [16], and fatal myocardial infarction [19]. However, increases in specific ceramide species contributing to the overall total concentration of plasma ceramides must be considered when determining metabolic disease and cardiovascular disease risk due to plasma ceramide levels. We also observed that HbA1c levels negatively correlated with C20:0 plasma concentrations which supports the notion that higher C20:0 ceramides may reflect a healthier phenotype. Finally, the role of higher levels S1P is controversial in MetD [36, 37]. S1P modulates cellular function and its concentrations increase in the plasma of obese patients compared to lean subjects [38]. Higher concentrations of S1P appear to impede insulin signaling leading to insulin resistance [39, 40]. Our finding that African Americans without MetD had higher concentrations of S1P may provide some insight into accelerated progression to an insulin resistant state in African Americans but also differences in dietary patterns.

Owing to the role of excess caloric consumption in ceramide concentration, it is not surprising that ceramides are elevated in persons with increased adiposity [41]. Interestingly, we did not observe a correlation between BMI and ceramide or sphingosine species (S1 Fig). One explanation for this observation may be lower ceramide concentrations in African Americans with MetD compared to other groups. Still, differences in BMI do not account for varying ceramide concentrations in our study cohort. Ceramides concentrations may provide mechanistic insight into racial disparities in insulin resistance and type 2 diabetes complications. Although our sphingolipid data did not correlate with serum creatinine concentrations (S2 Fig), previous studies suggest that C16:0, C20:0, C24:0 and C24:1-ceramide concentrations represent a promising prognostic tool for predicting diabetes-induced disease processes such as nephropathy [42]. Thus, inclusion of rapid ceramide analysis at the time of diabetes diagnosis could serve as a useful prognostic tool for early intervention to predict and prevent additional pathophysiological processes that commonly follow metabolic disorders.

Although offering new insights into the differential expression of ceramides in African Americans and whites based on metabolic disorder status, this study has several limitations. As stated previously, this is a small pilot study with a limited number of research subjects included; therefore, we cannot definitively conclude that differential expression in specific ceramide species persist based on metabolic disease status and/or race. Similarly, the small sample sizes precluded us from investigating whether the noted differences were confounded by other patient characteristics such as age, smoking status, or socioeconomic factors. A larger sample size is needed to determine the role of potential confounders. A recent study using a large cohort of patients from the Jackson Heart Study examined various ceramides and found that race influenced ceramide expression [43]; however, this study examined different ceramide species in relationship to visceral adiposity and was specifically focused on baseline differences in ceramide species amongst healthy controls free of diabetes and cardiovascular disease. Future studies will focus on inclusion of a larger sample size in order to validate our results.

Our data are further limited by our inability to validate the connection between plasma concentrations of ceramides and skeletal muscle tissue concentrations of ceramides. Anti-hypertensive and cholesterol lowering medication use were self-reported and we did not have data on compliance or the degree of control for the treated condition. Moreover, it is well recognized that cholesterol-lowering medications including simvastatin and rosuvastatin lower plasma ceramide levels and due to the small sample size of the cohort, we were unable to adjust for the use of these medications [44, 45]. In future studies, we will include a larger number of study subjects to expand this work. Lastly, we did not account for confounders that might contribute to differences in plasma ceramide concentrations such as dietary habits. Our study serves as a foundation for establishing baseline levels of ceramides in African American populations with and without MetD.

## Conclusion

In conclusion, we demonstrated that total plasma ceramide levels were lower in African Americans with MetD compared to African Americans without MetD. Specifically, we found a decrease in the plasma concentration of the very long chain C20 ceramide in African Americans with MetD compared with whites with MetD. Furthermore, very long chain ceramide species, C24:0, and C24:1 ceramides are decreased African Americans with MetD compared with African Americans without MetD. The plasma levels of C20 ceramide inversely correlates with HbA1c levels. Overall, our data suggest that ceramide metabolism is differentially regulated in African Americans with MetD and this may contribute to disparities observed in health outcomes. These results establish the need to conduct additional plasma ceramide studies in ethnically diverse groups to improve the global clinical utility of ceramides as biomarkers for CVD and metabolic disease risk estimation.

## Supporting information

S1 Fig Variations in BMI do not correlate with differential ceramide or sphingolipid concentrations amongst groups. Correlation of BMI and (A) ceramide C16:0 (B) ceramide C20:0, (C) ceramide C24:0, (D) ceramide C24:1, (E) total ceramides, (F) Sph-1p. Statistical analysis is based on Pearson r correlation statistic.
(TIFF)

S2 Fig Variations in serum creatinine concentrations do not correlate with differential ceramide or sphingolipid levels amongst groups. Correlation of serum creatinine concentrations and (A) ceramide C16:0 (B) ceramide C20:0, (C) ceramide C24:0, (D) ceramide C24:1, (E) total ceramides, (F) Sph-1p. Statistical analysis is based on Pearson r correlation statistic.
(TIFF)
