# Supplementary figures and images for: Differences in plasma levels of long chain and very long chain ceramides between African Americans and whites: An observational study
Source: PLoS One. 2019 May 8;14(5):e0216213. doi: 10.1371/journal.pone.0216213 (PMC6505935; doi:10.1371/journal.pone.0216213)

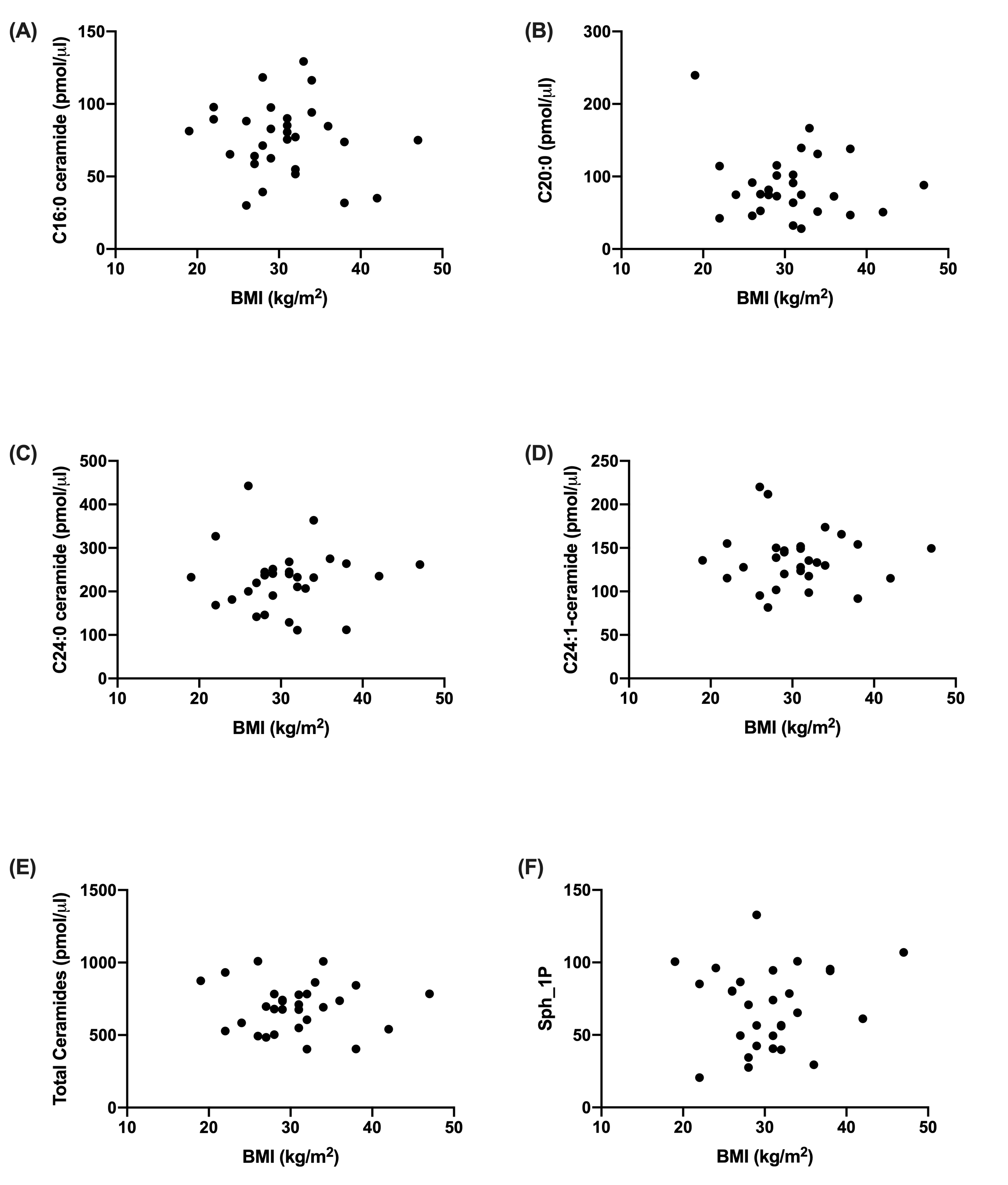

Supplement: S1 Fig — Correlation of BMI and (A) ceramide C16:0 (B) ceramide C20:0, (C) ceramide C24:0, (D) ceramide C24:1, (E) total ceramides, (F) Sph-1p. Statistical analysis is based on Pearson r correlation statistic. (TIFF) [file pone.0216213.s001.tiff]

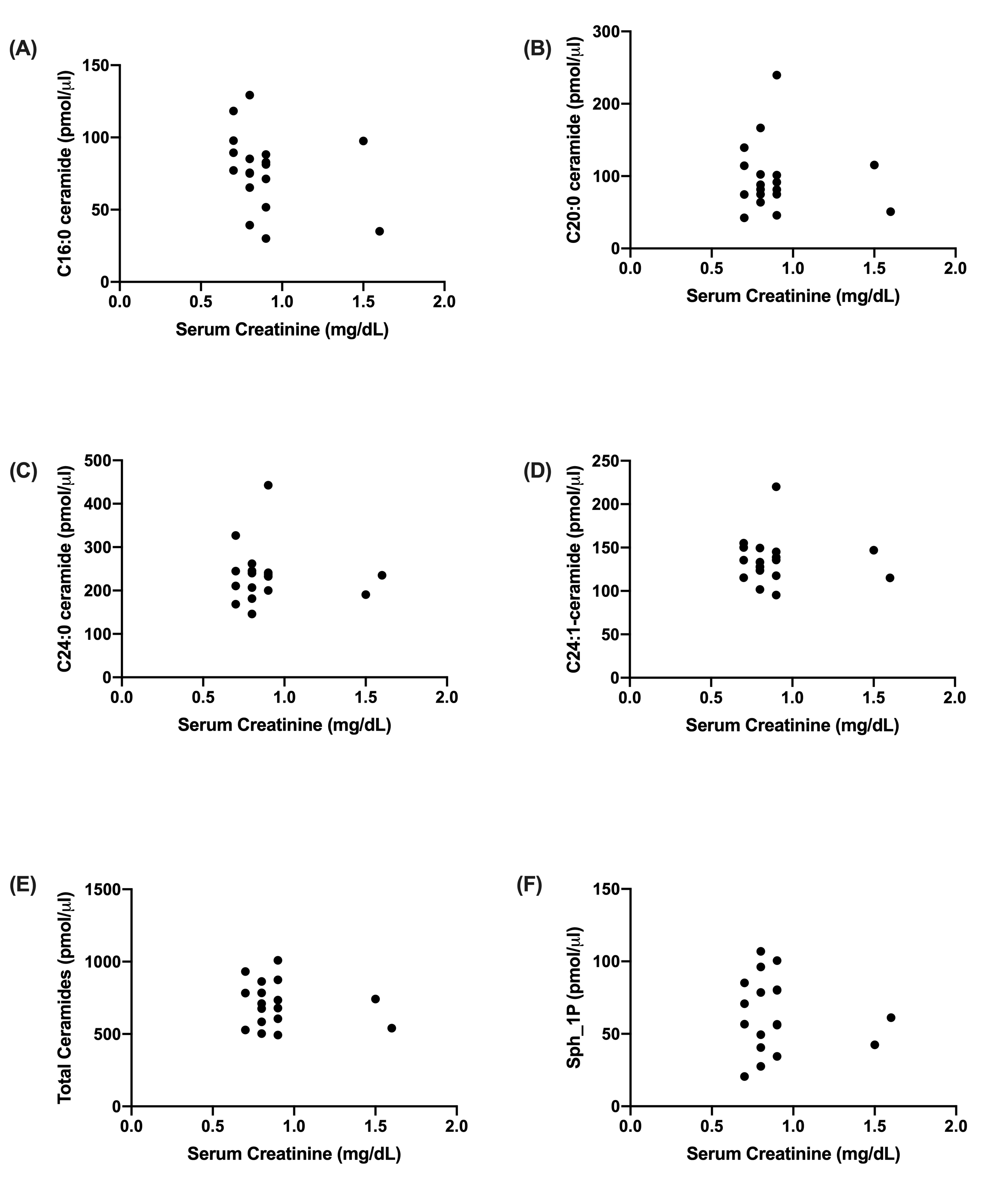

Supplement: S2 Fig — Correlation of serum creatinine concentrations and (A) ceramide C16:0 (B) ceramide C20:0, (C) ceramide C24:0, (D) ceramide C24:1, (E) total ceramides, (F) Sph-1p. Statistical analysis is based on Pearson r correlation statistic. (TIFF) [file pone.0216213.s002.tiff]
